# Socioeconomic status determines COVID-19 incidence and related mortality in Santiago, Chile

**DOI:** 10.1126/science.abg5298

**Published:** 2021-04-27

**Authors:** Gonzalo E. Mena, Pamela P. Martinez, Ayesha S. Mahmud, Pablo A. Marquet, Caroline O. Buckee, Mauricio Santillana

**Affiliations:** 1 Department of Statistics, University of Oxford, Oxford, UK.; 2 Center for Communicable Disease Dynamics, Department of Epidemiology, Harvard T.H. Chan School of Public Health, Boston, MA, USA.; 3 Department of Microbiology, University of Illinois at Urbana-Champaign, Urbana, IL, USA.; 4 Department of Statistics, University of Illinois at Urbana-Champaign, Champaign, IL, USA.; 5 Carl R. Woese Institute for Genomic Biology, University of Illinois at Urbana-Champaign, Urbana, IL, USA.; 6 Department of Demography, University of California, Berkeley, CA, USA.; 7 Departamento de Ecología, Facultad de Ciencias Biológicas, Pontificia Universidad Católica de Chile, Santiago, Chile.; 8 Instituto de Ecología y Biodiversidad (IEB), Santiago, Chile.; 9 The Santa Fe Institute, Santa Fe, NM, USA.; 10 Instituto de Sistema Complejos de Valparaíso (ISCV), Valparaíso, Chile.; 11 Centro de Cambio Global UC, Pontificia Universidad Católica de Chile, Santiago, Chile.; 12 Computational Health Informatics Program, Boston Children’s Hospital, Boston, MA, USA.; 13 Department of Pediatrics, Harvard Medical School, Boston, MA, USA.

## Abstract

The COVID-19 pandemic has affected cities particularly hard. Here, we provide an in-depth characterization of disease incidence and mortality and their dependence on demographic and socioeconomic strata in Santiago, a highly segregated city and the capital of Chile. Our analyses show a strong association between socioeconomic status and both COVID-19 outcomes and public health capacity. People living in municipalities with low socioeconomic status did not reduce their mobility during lockdowns as much as those in more affluent municipalities. Testing volumes may have been insufficient early in the pandemic in those places, and both test positivity rates and testing delays were much higher. We find a strong association between socioeconomic status and mortality, measured by either COVID-19–attributed deaths or excess deaths. Finally, we show that infection fatality rates in young people are higher in low-income municipalities. Together, these results highlight the critical consequences of socioeconomic inequalities on health outcomes.

## Urban socioeconomics and mortality

Santiago, Chile, is a highly segregated city with distinct zones of affluence and deprivation. This setting offers a window on how social factors propel the severe acute respiratory syndrome coronavirus 2 (SARS-CoV-2) pandemic in an economically vulnerable society with high levels of income inequality. Mena *et al.* analyzed incidence and mortality attributed to SARS-CoV-2 to understand spatial variations in disease burden. Infection fatality rates were higher in lower-income municipalities because of comorbidities and lack of access to health care. Disparities between municipalities in the quality of their health care delivery system became apparent in testing delays and capacity. These indicators explain a large part of the variation in COVID-19 underreporting and deaths and show that these inequalities disproportionately affected younger people.

*Science*, abg5298, this issue p. eabg5298

## Abstract (structured)

**INTRODUCTION:**

The COVID-19 crisis has exposed major inequalities between communities. Understanding the societal risk factors that make some groups particularly vulnerable is essential to ensure more effective interventions for this and future pandemics. Here, we focus on socioeconomic status as a risk factor. Although it is broadly understood that social and economic inequality has a negative impact on health outcomes, the mechanisms by which socioeconomic status affects disease outcomes remain unclear. These mechanisms can be mediated by a range of systemic structural factors, such as access to health care and economic safety nets. We address this gap by providing an in-depth characterization of disease incidence and mortality and their dependence on demographic and socioeconomic strata in Santiago, a highly segregated city and the capital of Chile.

**RATIONALE:**

Combining publicly available data sources, we conducted a comprehensive analysis of case incidence and mortality during the first wave of the pandemic. We correlated COVID-19 outcomes with behavioral and health care system factors while studying their interaction with age and socioeconomic status. To overcome the intrinsic biases of incomplete case count data, we used detailed mortality data. We developed a parsimonious Gaussian process model to study excess deaths and their uncertainty and reconstructed true incidence from the time series of deaths with a new regularized maximum likelihood deconvolution method. To estimate infection fatality rates by age and socioeconomic status, we implemented a hierarchical Bayesian model that adjusts for reporting biases while accounting for incompleteness in case information.

**RESULTS:**

We find robust associations between COVID-19 outcomes and socioeconomic status, based on health and behavioral indicators. Specifically, we show in lower–socioeconomic status municipalities that testing was almost absent early in the pandemic and that human mobility was not reduced by lockdowns as much as it was in more affluent locations. Test positivity and testing delays were much higher in these locations, indicating an impaired capacity of the health care system to contain the spread of the epidemic. We also find that 73% more deaths than in a normal year were observed between May and July 2020, and municipalities at the lower end of the socioeconomic spectrum were hit the hardest, both in relation to COVID-19–attributed deaths and excess deaths. Finally, the socioeconomic gradient of the infection fatality rate appeared particularly steep for younger age groups, reflecting worse baseline health status and limited access to health care in municipalities with low socioeconomic status.

**CONCLUSION:**

Together, these findings highlight the substantial consequences of socioeconomic and health care disparities in a highly segregated city and provide practical methodological approaches useful for characterizing the COVID-19 burden and mortality in other urban centers based on public data, even if reports are incomplete and biased.

## Teaser

The consequences of health care inequalities have been exacerbated during the SARS-CoV-2 pandemic in a large, economically vulnerable city.

## Graphical abstract

**Figure:**
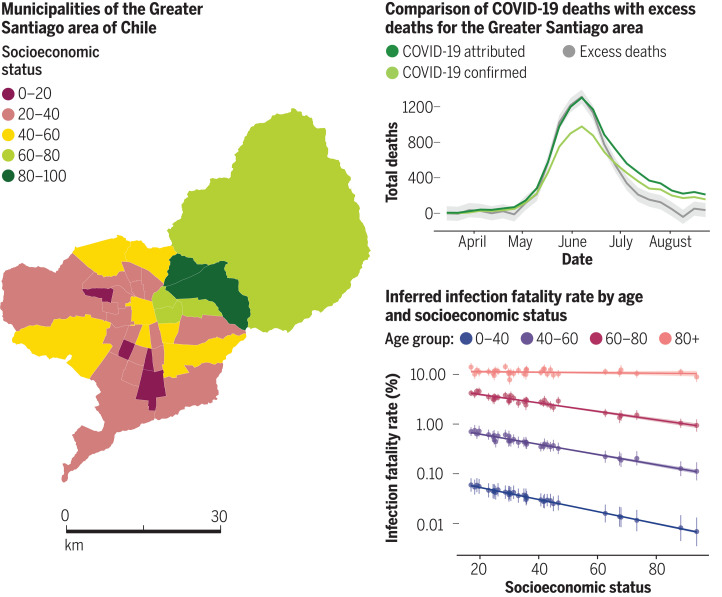
Effect of socioeconomic inequalities on COVID-19 outcomes. The map on the left shows the municipalities that were included in this study, colored by their socioeconomic status score. For the comparison between COVID-19 deaths and excess deaths (top right), COVID-19–confirmed deaths are shown in light green and COVID-19–attributed deaths in dark green. Excess deaths, shown in gray, correspond to the difference between observed and predicted deaths. Predicted deaths were estimated using a Gaussian process model. The shading indicates 95% credible intervals for the excess deaths. The infection fatality rates (bottom right) were inferred by implementing a hierarchical Bayesian model, with vertical lines representing credible intervals by age and socioeconomic status.

The COVID-19 pandemic is an ongoing public health crisis. Although many studies have described the transmission of severe acute respiratory syndrome coronavirus 2 (SARS-CoV-2)—the virus that causes COVID-19—in North America, Europe, and parts of Asia (*1*–*5*), the characterization of the pandemic in South America has received less attention, despite the severe impact in many countries during the Southern Hemisphere winter season. Although confirmed COVID-19 cases are an important public health measure to estimate the level of spread of infections caused by SARS-CoV-2, they may not be a reliable indicator of incidence because of biases due to population-level health-seeking behavior, surveillance capacities, and the presence of asymptomatic individuals across regions (*6*). Analyses of COVID-19–related deaths as well as excess mortality provide an alternative and potentially less biased picture of epidemic intensity (*7*, *8*). This is in part because ascertainment biases may be less pronounced for deaths than for confirmed cases, because people dying from COVID-19 are more likely to have experienced severe symptoms and thus are more likely to have been documented as COVID-19–positive cases by health surveillance systems. Age-specific death data may also help explain the heterogeneity in COVID-19 burden and COVID-19–attributable deaths in different countries (*9*). However, the role of other factors—such as socioeconomic status, which is correlated with health care access—on fatality and disease burden remains a particularly important open question (*10*) for cities with substantial economic disparities.

Here, we analyzed incidence and mortality attributed to SARS-CoV-2 infection and its association with demographic and socioeconomic status across the urban metropolitan area of the capital of Chile, known as “Greater Santiago.” Unlike many other countries, Chile set up a notably thorough reporting system and made many key datasets publicly available. To understand spatial variations in disease burden, we estimated excess deaths and infection fatality rates across this urban area. To understand disparities in the health care system, we analyzed testing capacity and delays across municipalities. We then demonstrate strong associations of these health indicators with demographic and socioeconomic factors. Together, our results show that socioeconomic disparities explain a large part of the variation in COVID-19 deaths and underreporting and that those inequalities disproportionately affected younger people.

## Association between socioeconomic status and disease dynamics

**Fig. 1:**
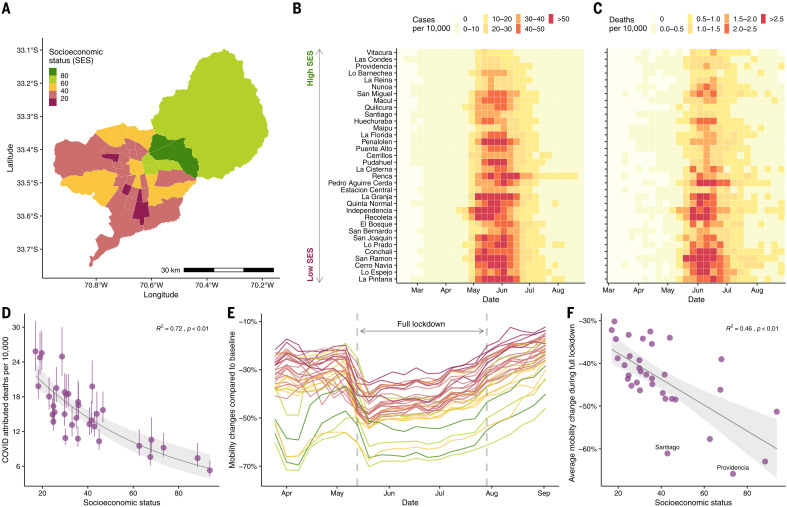
SES, COVID-19 cases and deaths, and mobility data in Greater Santiago. (**A**) Municipalities that are part of Greater Santiago are colored according to their SES, where a lower score is indicative of a lower SES. (**B**) COVID-19 cases normalized by population size per municipality. Municipalities are sorted by SES, starting with the one that has the highest SES at the top. (**C**) COVID-19–attributed deaths normalized by population size per municipality. (**D**) Age-adjusted COVID-19–attributed death rate and its association with SES. The dots and the whiskers represent the median and the 95% confidence intervals, respectively, reflecting uncertainty on the standard population used for weighting. *R*^2^, coefficient of determination. (**E**) Daily reduction in mobility by municipality colored by its SES value. (**F**) Average reduction in mobility during the full lockdown period and its association with SES. The urban and the business centers, Santiago and Providencia, respectively, experienced a greater reduction in mobility than expected based just on their socioeconomic profile. In (D) and (F), the shaded area indicates 95% regression confidence interval.

The Greater Santiago area is composed of 34 municipalities—defined as having more than 95% of its area urbanized—and is home to almost 7 million people. Although this urban center accounts for 36% of the country’s population, it has reported 55% of the confirmed COVID-19 cases and 65% of the COVID-19–attributed deaths before epidemiological week 36 (end of August 2020). Socioeconomic status (SES) in the municipalities varies widely, with Vitacura having the highest value (SES = 93.7) and La Pintana the lowest (SES = 17.0; Fig. 1A), and this difference is reflected in the impact of the pandemic during the Southern Hemisphere winter of 2020. The maximum incidence in Vitacura was 22.6 weekly cases per 10,000 individuals during the middle of May, whereas La Pintana reported a maximum of 76.4 weekly cases per 10,000 individuals during the first week of June (Fig. 1B). As shown in Fig. 1C and fig. S1, the attributed COVID-19 deaths follow a similar (yet lagged) temporal pattern to the number of reported COVID-19 cases. For instance, the highest rate of 4.4 weekly deaths per 10,000 individuals is observed in San Ramon, a municipality with a SES of 19.7, whereas Vitacura reported a maximum of 1.6 weekly deaths per 10,000 in June. These social inequalities affect the overall COVID-19 mortality rates, as shown in Fig. 1D.

Changes in human mobility—a proxy for physical distancing—during lockdown periods follow a similar trend. Using human mobility indicators, inferred from anonymized mobile phone data obtained from the Facebook Data for Good Initiative, we show that the two municipalities with highest SES exhibited a reduction in mobility by up to 61% during the full lockdown (dark green regions in Fig. 1E) compared with the ones with lowest SES, which, on average, reduced their mobility to 40% during the this period (dark pink regions in Fig. 1E). This relationship between reductions in mobility and SES was present during all time periods considered for this study (Fig. 1F) and supports the hypothesis that people in poorer regions cannot afford to stay at home during lockdowns. Our result is consistent with analyses of New York City neighborhoods (*11*) and with findings from other studies conducted in Santiago that used different socioeconomic and mobility metrics (*12*–*14*).

## Epidemic reconstruction reveals early transmission dynamics

**Fig. 2:**
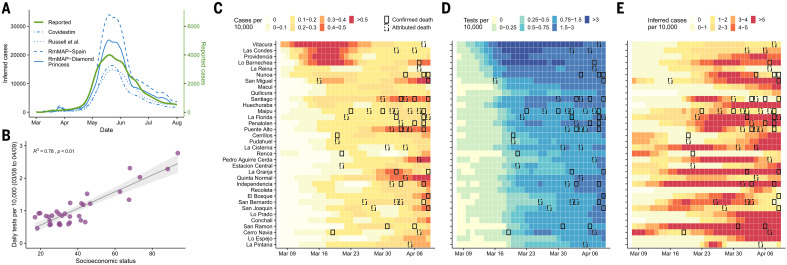
Inferred cases and reported tests conducted for the Greater Santiago area. (**A**) Inferred and reported cases over time. For our RmMAP reconstructions, we considered the log-normal onset-to-death distribution described in (*38*) and two age-stratified IFR estimates, one from the Diamond Princess cruise ship (*39*) and another from a seroprevalence study in Spain (*40*). For comparison, we also present reconstructions based on the Covidestim method (*41*) and by the rescaling of case counts by the underreporting of estimates obtained with the method of Russell *et al*. (*42*). (**B**) Association between average daily tests and SES during the early peak. The early peak is defined as those cases reported between 8 March and 4 April. The shaded area indicates 95% regression confidence interval. (**C**) Reported cases per 10,000 by municipality during the early peak. (**D**) Tests per 10,000 by municipality during the early peak. (**E**) Inferred cases obtained from the RmMAP-Spain model per 10,000 by municipality during the early peak. For (C) to (E), the record of at least one COVID-19–confirmed or COVID-19–attributed death for that particular week is highlighted with solid or dashed boxes, respectively.

To examine the possible bias present in the incidence data, we reconstructed SARS-CoV-2 infections over time by implementing a method called regularized mortality MAP (RmMAP). RmMAP back-calculates the most likely infection numbers given the temporal sequence of deaths, the onset-to-death distribution, and the demography-adjusted infection fatality rate (IFR). Figure 2A shows the outcomes of this inference process, where the reconstructions from our approach and other methods are able to capture the main peak observed in May and June, with an estimate of the number of infected individuals that is 5 to 10 times greater than the reported values.

The reconstructions also reveal important differences in the inferred number of infections during March 2020, the month in which the virus was introduced in Chile by travelers from affluent municipalities. We analyzed the number of tests performed between 8 March and 9 April and find a significantly higher number of tests performed in municipalities with high SES (Fig. 2B), especially during the first 2 weeks of March (Fig. 2D). In addition, an early peak of reported cases was only observed in high-SES municipalities during the middle of March (Fig. 2C), even though several COVID-19 deaths, which are lagged with respect to infection by up to several weeks, were reported in low-SES municipalities during the same period. These findings suggest that an early first wave of infections occurred during March and quickly spread through the rest of the city without being captured by the official counts. Our RmMAP estimates at the municipality level support this claim, because they capture a high volume of early infections in most municipalities (Fig. 2E), a scenario that largely deviates from the official tallies (Fig. 2C).

To further validate the hypothesis of an early underreporting in low-SES municipalities, and to rule that these early activity estimates are not an artifact of our method, we performed experiments on a synthetic elementary model of two peaks of different sizes separated in time (supplementary materials). These experiments confirm that RmMAP is capable of recovering this bimodal phenomena, whereas other methods fail to do so; they oversmooth the true signal, and the earlier peak is typically not recovered. This early underreporting signal suggests that the patterns of mortality and testing observed across Greater Santiago are partially explained by an early failure of health care systems in informing the population with sufficient situational awareness about the real magnitude of the threat (*15*).

## Excess deaths match COVID-19–attributed deaths

**Fig. 3:**
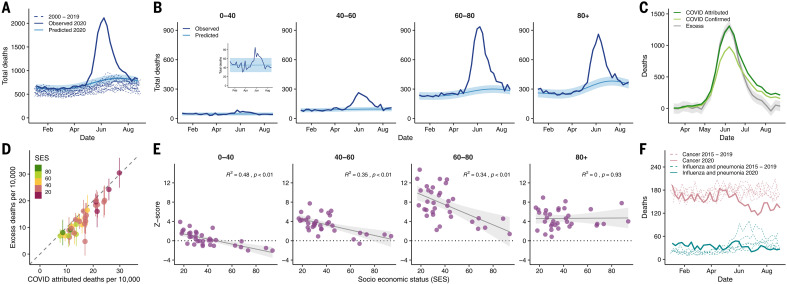
Excess deaths and its association with demographic and socioeconomic factors. (**A**) Observed deaths (solid dark blue line) in Greater Santiago compared with predicted deaths for 2020 (solid light blue line and its confidence intervals shaded in lighter color), using a Gaussian process regression model built with historical mortality data from 2000 to 2019 (dashed blue lines). The values contain all the possible causes of deaths. (**B**) Age-specific trends of the observed deaths compared with the predicted deaths for 2020. (**C**) COVID-19 deaths versus excess deaths. COVID-19–confirmed deaths are shown in light green, whereas COVID-19–attributed deaths are shown in dark green. Excess deaths correspond to the difference between observed and predicted deaths. (**D**) Comparison of excess deaths and COVID-19–attributed deaths per municipality colored by SES and normalized by population size. (**E**) Monthly average of *z* scores of observed deaths between April and July by age group. The *z* scores correspond to the standard deviations over expected values. (**F**) Historical deaths due to influenza and pneumonia (teal dashed lines) and cancer (pink dashed lines) compared with the observed deaths during 2020 (solid lines). In (B), (C), and (E), the shaded region indicates 95% regression confidence interval.

Excess deaths, defined as the difference between observed and expected deaths, can provide a measure of the actual impact of the pandemic in mortality by quantifying direct and indirect deaths related to COVID-19 (*7*, *8*, *16*). We estimated the expected deaths for 2020 by fitting a Gaussian process model (*17*) to historical mortality data from the past 20 years and used them to identify the increased mortality during the pandemic, controlling for population growth and seasonality. As shown in Fig. 3A, the number of deaths observed between May and July 2020 is more than 1.73 (confidence interval 1.68 to 1.79) times the expected value, with a peak surpassing 2110 death counts in epidemiological week 24 (first week of June 2020) compared with an expected value of 802 deaths and an average number of deaths of 798 between 2015 and 2019.

When comparing the number of deaths by age in the year 2020 with our model’s predictions, we observe pronounced patterns. Although people younger than 40 years old have an overall lower mortality rate than those from older age groups as expected, they still exhibit a nearly twofold increase in the total deaths, with a peak in the observed deaths occurring 2 weeks earlier than that for those older than 60 years of age (Fig. 3B). For the age groups 40 to 60, 60 to 80, and older than 80, the observed deaths are 2.8, 3.2, and 2.4 times higher than expected, respectively. Even though the age group 80+ exhibits the highest expected mortality values for 2020, the group that contains people between 60 and 80 years old displays the highest weekly count (936 during epidemiological week 24), the biggest deviation from the predicted values, and the highest values of excess deaths (645 more deaths than expected; Fig. 3B).

COVID-19–attributed deaths for the entire Greater Santiago area fall withing the credible intervals of excess deaths until late June, when the attributed deaths increase to rates that are even higher than the excess deaths, suggesting that underreporting in COVID-19–attributed deaths is unlikely (Fig. 3C). COVID-19–confirmed deaths—those with a polymerase chain reaction (PCR)–confirmed SARS-CoV-2 test—follow a similar temporal pattern, and the difference between confirmed and COVID-19–attributed deaths gets smaller toward the end of August, indicative of an improved testing capacity. This pattern is consistent when compared with normalized deaths by population size for each municipality (Fig. 3D), which also shows COVID-19–attributed deaths higher than the excess deaths in most of the cases. The anomalies in the observed versus predicted deaths for 2020 across different age groups also display a significant negative association with SES, except for the 80+ group (Fig. 3E), suggesting a higher death burden in lower-SES municipalities, independent of their age composition. Furthermore, the two municipalities with a SES higher than 80 (Las Condes and Vitacura) had *z* scores of much smaller magnitude (with the exception of the oldest age group), indicating that in those areas, patterns of mortality did not deviate much from what would have been expected for a normal year in people younger than 80 years old.

Although the observation that COVID-19–attributed deaths are greater than the estimated excess deaths might be counterintuitive (Fig. 3D), it may indicate the presence of changes in overall mortality patterns due to other causes, including a lower number of deaths attributable to a reduction in mobility. In addition, lower numbers of deaths were reported for respiratory infectious diseases, such as influenza and pneumonia, and cancer during July and August 2020 compared with the period from 2015 to 2019 (Fig. 3F). Changes in mortality from respiratory diseases can be explained by a mild influenza season in the Southern Hemisphere during the winter of 2020 (*18*), which is consistent with our observation that far fewer cases of respiratory viruses have been detected in Chile during the 2020 season (supplementary materials). A decrease in the number of cancer-attributed deaths can be explained by mortality displacement (*19*, *20*), but additional analyses need to be conducted to establish this hypothesis. Alternative explanations for changes in all-cause mortality should also consider possible changes in external and behavioral causes of mortality. We do not observe a substantial contribution from these causes (see supplementary materials, along with additional detailed analyses).

## More testing with lower waiting times in wealthy areas

**Fig. 4:**
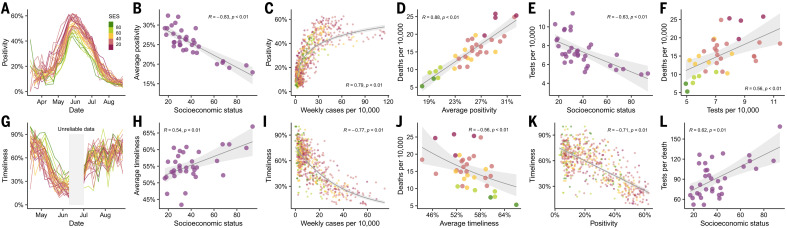
Testing capacity and waiting times. (**A**) Positivity over time. Positivity is defined as the proportion of PCR tests that are positive in a given week. (**B**) Association between average positivity and SES. (**C**) Association between positivity and weekly number of cases per 10,000. (**D**) Association between the overall age-adjusted number of deaths per 10,000 and the average positivity over the same period. (**E**) Association between average daily tests per 10,000 and SES. (**F**) Association between tests per 10,000 and deaths per 10,000. (**G**) Timeliness over time. Timeliness is defined as the proportion of PCR tests that appear in the public records within 1 week from the onset of symptoms. Two weeks in June (shaded in gray) were excluded from the analysis because of inconsistencies in data, leading to unreliable delay estimates. (**H**) Association between average timeliness and SES. (**I**) Association between timeliness and weekly number of cases per 10,000. (**J**) Association between the overall age-adjusted number of deaths per 10,000 and the average timeliness. (**K**) Association between timeliness and positivity. Dots are representative of weekly data per municipality. (**L**) Association between tests per death (age-adjusted) and SES. Figures with different dot colors illustrate the SES value according to the reference presented in (A). In (B) to (F) and (H) to (L), the shaded region indicates 95% regression confidence interval.

To further understand the consequences of insufficient early testing, we conducted a deeper analysis of different testing metrics at the municipality level. We first looked at testing capacity measured as weekly positivity rates, the fraction of tests that are positive for SARS-CoV-2. Our results show that the positivity signal tracked the course of the epidemic, peaking at times of highest incidence between May and July and suggesting a highly saturated health care system during this period across the entire city (Fig. 4A). A strong negative association between positivity and SES (Fig. 4B) further denotes difficulties in access to health care that is even more pronounced in lower-SES municipalities. Despite changes in positivity rates over time, this testing metric also significantly correlated with the number of cases (Fig. 4C) and number of deaths (Fig. 4D).

Our findings on the number of tests conducted show a rather paradoxical association with SES and mortality. Many months into the epidemic, the early positive association between tests per capita and SES (Fig. 2B) reversed (Fig. 4E), indicative of an improvement in testing capacity over time, so that more tests were performed in the most affected areas. Similarly, the number of tests started to positively correlate with deaths (Fig. 4F), suggesting that the number of tests are strong predictors of mortality.

We also analyzed testing capacity by estimating the delays in obtaining test results. We inferred the distribution of the delay between onset of symptoms and report of the results, from which we obtained the proportion of cases that are publicly reported within 1 week of the onset of symptoms or “timeliness” (*21*). As shown in Fig. 4G, timeliness follows a similar temporal course as test positivity during May and part of June, but in the opposite direction. This metric is associated with SES, suggesting that municipalities with low SES, on average, get their test results later than the ones with high SES (Fig. 4H). Timeliness also negatively correlates with the number of cases (Fig. 4I), the total number of deaths (Fig. 4J), and positivity (Fig. 4K). When looking at tests per death, a metric that can be used as a faithful proxy of testing capacity (*22*), we observe a positive correlation with SES (Fig. 4L), indicating that testing disparities persisted during the epidemic, with low-SES areas being affected the most. In the supplementary materials, we further discuss the associations between our metrics and case counts.

## IFR depends on SES

**Fig. 5:**
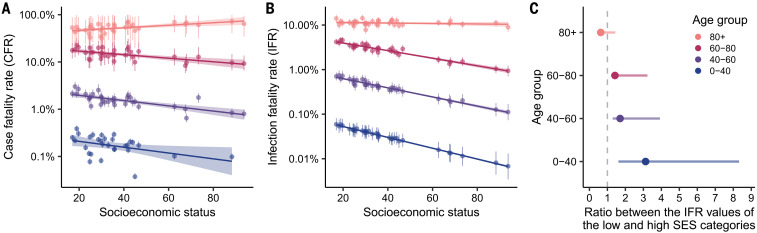
Inference of CFRs and IFRs by age and SES. (**A**) Estimates of CFR by age and SES based on a simple assignment of cases to age groups. Our estimates of CFR have been validated by the official ICOVID platform (www.icovidchile.cl/), which confirmed that 119 out of the 136 observed CFRs fall within our confidence intervals. Confidence intervals are derived from a bootstrap procedure described in the supplementary materials. (**B**) Inferred IFR by age and SES using our ensemble of hierarchical Bayesian models, along with associated credible intervals. (**C**) IFR ratio between the low- and high-SES categories by age group. Four socioeconomic categories were defined based on SES quantiles: low, mid-low, mid-high, and high.

In the absence of serological surveys, a direct inference of an IFR is challenging. The degree of ascertainment depends on many factors, including testing capabilities and the likelihood of having symptomatic infections. Also, unlike deaths, age information of reported cases is not available at the municipality level, making this inference more challenging. To address these hurdles and to have estimates of the IFR, we implemented a hierarchical Bayesian model that considers the relationship between deaths, observed cases, and true infections across location, time, and age group. We first estimated the case fatality rate (CFR) by assigning total cases into age groups in a simple way that projects the overall age distribution of cases to particular municipality demographics (Fig. 5A; see supplementary materials for details). With the exception of the oldest age group, CFR shows a negative association with SES. Similarly, our resulting IFR estimates once corrected for underascertainment display a similar pattern (Fig. 5B) but on an order of magnitude lower than the CFR estimates. We then grouped the municipalities into four categories of similar sizes and categorized them as either low, mid-low, mid-high, or high SES. When comparing the IFR ratio between the low- and the high-SES categories, the results show a significantly higher IFR in the low-SES group in people younger than 80 years old (Fig. 5C). The age groups 60 to 80 and 40 to 60 exhibit IFRs that are 1.4 and 1.7 times higher, respectively, in the low-SES category compared with the high-SES one. The difference is even more pronounced in the younger age group (0 to 40 years old), which shows values of IFR that are 3.1 times higher for the municipalities with the lowest SES. Altogether, these results are in line with the analyses of excess deaths presented in Fig. 3E. The lack of association between IFR and SES in the oldest age group can be attributed to a lower life expectancy (*23*), which is factored into the estimation of SES (see methods for details), and the fact that elderly people might be, in general, healthier enough to survive until that age.

## Discussion

To understand the true burden of COVID-19, it is critical to consider demographic and socioeconomic factors and their consequences for the public health response. Here, we analyzed data from the capital of Chile, a highly segregated city. Our results align with the recent literature on uneven health risks globally, which has highlighted how socially and economically deprived populations are more vulnerable to the burden of epidemics (*24*, *25*). Mounting evidence suggests that such differences have also manifested in the context of the COVID-19 pandemic (*26*, *27*). Because the pathways modulating these differential outcomes are not well understood, comprehensive accounts are urgently needed (*28*) so that more resilient and socially aware public health strategies can be planned in advance of future pandemics. In Chile, recent studies have suggested a link between SES and the effectiveness of nonpharmaceutical interventions such as stay-at-home orders (*12*, *13*, *29*). Our work further explores this topic by providing a holistic perspective about how the interplay between behavioral, social, economic, and public health factors modulates the observed heterogeneity in infection incidence and mortality. Along with the main findings, we also introduced several methodological innovations. Our Bayesian method for joint inference of IFRs and underreporting is a new contribution in this field. We show that it may not be necessary to have complete epidemiological datasets (here, age) to draw valid inferences as long as the solution space is constrained enough by meaningful priors and demographic structure.

Our results show a strong link between socioeconomic and demographic factors with COVID-19 outcomes and testing capacity of COVID-19 in Santiago. This association is manifested as a reinforcing feedback loop, as highlighted by our findings. First, our analysis of human mobility indicates that municipalities with lower SES were less compliant with stay-at-home orders, possibly because people from lower-SES areas are unable to work from home, which leaves them at a higher disease risk. Second, our analyses revealed an underreporting of infections in low-income areas at the start of the outbreak. Because public health measures were taken in response to nominal case counts, these places were underprepared, with a poor health care response that resulted in higher death counts. Third, anomalies in the overall excess deaths are higher in low-SES areas, particularly in people younger than 80 years old, suggesting that more vulnerable municipalities were hit the hardest. Fourth, the analyses of test positivity rates, timeliness, and tests per death indicate an insufficient deployment of resources for epidemiological surveillance. Higher positivity rates in health care centers suggest the need for greater testing and detection. At the same time, slower turnaround in test results can lead to greater potential for transmission, because even small delays between the onset of symptoms, testing, and final isolation substantially hinder the capability of public health systems to contain the epidemic (*30*). Finally, IFRs were higher in lower-SES municipalities, especially among younger people.

We propose two complementary explanations for the association between IFR and SES. First, a higher IFR may reflect limited access to health services during the pandemic, and the strong association between the number of tests per death and SES supports this claim. We also show in the supplementary materials that the South and West zones (based on health coverage division) have four times fewer beds per 10,000 people and four times lower proportion enrolled in the private health system than the East zone, which contains all the municipalities with an SES of 60 or higher. Notably, more than 90% of the COVID-19–attributed deaths in the South and West zones occurred in places other than health care facilities, compared with 55% in the East zone. Second, more vulnerable communities may experience a higher prevalence of the comorbidities (*31*) that are associated with more severe presentations of COVID-19. People in low-SES municipalities are more likely to be overweight and to live in overcrowded conditions (supplementary materials), factors that ultimately can put these populations at higher disease risk. The interaction of these two explanations can lead to a high disparity among different socioeconomic groups.

Our findings need to be considered in light of the following limitations. Mobility data from mobile phones are likely to be biased because of differential mobile phone ownership in different demographic groups. Although Facebook mobility data can be biased in this way, our results are consistent with other studies in Santiago that used different socioeconomic and movement measurements [see (*12*–*14*) and supplementary materials]. Our methods depend on several assumptions. The back-calculated RmMAP estimates rely on a choice of the infection-to-death distribution and assume that the IFRs do not change over time, and the excess mortality estimates depend on the choice of a kernel. Our IFR estimates are derived from a complex Bayesian model and are based on assumptions regarding reporting rates and age distribution of infections. Extensive sensitivity analyses suggest that our results are stable to deviations from these assumptions (supplementary materials).

This study highlights major consequences of health care disparities in a highly segregated city and provides new methodologies that account for incomplete data for studying infectious disease burden and mortality in other contexts.

## Materials and methods

### Data

#### SES

We define the socioeconomic status index (SES) as SES = 100 − SPI, where SPI is the social priority index (or “indice de prioridad social” in Spanish) estimated for 2019. The SPI index varies between 0 and 100 and has been reported yearly since 1995 by the Chilean Ministry of Social Development and Family. The SPI value denotes the priority of each municipality for the social programs of the regional government, and thus, municipalities with lower SES have higher social priority. The SPI index equally weights three components: (i) income and poverty, (ii) access and quality of education, and (iii) health factors such as access to health care and life expectancy. For each component, the values are standardized on a common scale from 0 to 100, where the value 100 represents the worst relative situation (highest priority) and 0 the best situation (least priority).

#### COVID-19

At the end of January 2020, the Chilean government determined that all suspected cases of COVID-19 must be notified in a mandatory and immediate manner to the respective Health Epidemiology Unit and the Ministry of Health, through the specific form on the EPIVIGILA platform. In addition to the suspected cases that are identified in health care facilities, the government also implemented an active testing surveillance program to identify asymptomatic and presymptomatic cases. The criteria for the active testing are (i) people who have not been identified yet as confirmed or suspected COVID-19 or (ii) living in vulnerable areas and (iii) individuals who have lived for a long time in institutions such as jails, nursing homes, the National Service for Minors, among others. Symptom-onset dates are reported by the patient to a physician, in the case that the person attended a health institution, or by the volunteers that are conducting surveillance in the community through a survey.

The Chilean Ministry of Science, Technology, Knowledge, and Innovation has made possible access to aggregated data collected through the EPIVIGILA platform, which are available in the format of multiple reports. These reports also contain data on population projections for 2020, testing, positivity, and other metrics used in the study. One of the reports tracks the number of cases for which onset of symptoms started at a given epidemiological week, for each municipality. Given that they are published twice a week (typically Monday and Friday), we were able to analyze the history of such reports to estimate the delays. Timeliness is thus defined as the probability of getting a retrospective delay smaller than 7 days, based on the Monday’s reports. More details can be found in the supplementary materials.

#### Mortality

The Vital Statistics System in Chile is continuous, mandatory, and centralized. It is composed of the Civil Registry and Identification Service (CRIS), the National Institute of Statistics, and the Ministry of Health through the Department of Health Statistics and Information (DHSI). When a person dies, a medical death certificate is generated by the CRIS and distributed to health institutions. The mortality database is built with the death certificates, which are subjected to a rigorous validation process, to guarantee the reliability and validity of the information. The DHSI standardizes the clinical terms in the format of the International Statistical Classification of Diseases (ICD-10). Since March 2020, the DHSI has implemented the recommendations of the World Health Organization for coding the deaths resulting from COVID-19. In this study, confirmed COVID-19 deaths correspond to deaths for which the virus has been identified with a positive PCR test and have been coded as U07.1. Similarly, attributed COVID-19 deaths correspond to confirmed deaths and deaths in which the virus was not identified but were clinically diagnosed as a probable or suspected COVID-19 case and have been coded as U07.2.

#### Human movement

Facebook’s Data for Good has provided access to their Geoinsights portal in response to the COVID-19 crisis, from where it is possible to obtain aggregated data of their users (*32*). These datasets are anonymized and contain the information of Facebook users who have a smartphone with location services enabled. The movement vector from tile *i* to *j* (with *i* ≠ *j*) at time *t* is defined as the transition from the modal location *i* at the preceding 8-hour bin to the modal location *j* in the current 8-hour bin. Facebook also provides a baseline value, defined as the average number of users who transit from tile *i* to *j* at a given day of week and time of day during a baseline period. The baseline period corresponds to the 45 days before the initiation of the movement data for that particular location (for Chile, the data collection was initialized on 25 March 2020). Using this dataset, we calculated the percentage change compared with baseline for each *i* to *j* transition at a given 8-hour period and then estimated the average percentage change for each municipality and epidemiological week. We only used the starting location (municipality) for the average percentage change estimation. The size of the side of the tile is about 2.4 km.

### Models

#### Inference of SARS-CoV-2 infections with RmMAP

We aim to estimate the number of infected individuals over time $I_{s}$ given a series of observed COVID-19–attributed deaths $D_{t}$ and a known onset-to-death distribution *T*. We use a Poisson deconvolution model for deaths given *I* and *T*:

$$D_{t}|T,I∼Poisson\left(\sum_{s}T_{t-s}I_{s}\right)$$ (1)

where $T_{s}=P\left(T=s\right)$ is the probability that the onset-to-death time equals *s* days. Estimates of *I* maximizing Eq. 1 can be obtained with an expectation maximization algorithm (*6*, *33*–*35*), but the outcome is typically unstable (*36*). RmMAP overcomes this issue by adding a quadratic penalty to the log-likelihood. The iterations of RmMAP write as

$${\hat{I}}^{new}=\frac{1}{4\lambda }\sqrt{1+8\lambda I^{old}}-1$$ (2)

$$I_{s}^{new}={\hat{I}}_{s}^{new}\frac{1}{\sum_{t}T_{t-s}}\sum_{t}\frac{D_{t}T_{t-s}}{\sum_{s^{\prime }}T_{t-s^{\prime }}{\hat{I}}_{s^{\prime }}^{new}}$$ (3)

By scaling the final series $I^{new}$ by the inverse IFR, we obtain the inferred values of infected individuals over time. A detailed discussion of this method along with sensitivity analysis and comparison with existing methodology are presented in the in the supplementary materials.

#### Estimation of excess deaths

We used Gaussian processes (GP) regression (*17*) to estimates excess deaths for 2020. GPs can be understood as an infinite dimensional Bayesian regression: In the finite dimensional case, one fits $y_{i}=\sum_{i}w_{i}x_{i}+\epsilon_{i}$, where $\epsilon_{i}$ are Gaussian independent identically distributed errors, $x_{i}$ are covariates, and $w_{i}$ are coefficients sampled from a prior $p\left(w\right)$. Likewise, with GPs we fit $y_{i}=f\left(x_{i}\right)+\epsilon_{i}$ where *f* is a function sampled from a prior over function $p\left(f\right)$. GPs are appealing because the level of complexity is automatically adjusted by the complexity of data and because they are computationally tractable.

Priors over *f* are specified through a kernel *K*, which encodes the correlational structure of data so that $K\left(x,x\prime \right)$ is simply the “prior” covariance between $f\left(x\right)$ and $f\left(x,\prime \right)$. *K* depends on a finite number of unknowns θ (so $K=K_{\theta }$) that have to be inferred as well.

We used a GP to account for both long-term trends in mortality as well as seasonality. As in (*17*), we consider kernels of the form

$$K_{\theta }=K_{\theta }^{1}+K_{\theta }^{2}$$ (4)

where $K_{\theta }^{1}$ is an exponential kernel representing the long-term variation and is given by

$$K_{\theta }^{1}\left(x,x^{\prime }\right)=\theta_{1}^{2}\exp\left(-\frac{{\left(x-x^{\prime }\right)}^{2}}{2\theta_{2}^{2}}\right)$$ (5)

and $K_{\theta }^{2}$ is a periodic times exponential kernel representing seasonal variation

$$K_{\theta }^{2}\left(x,x^{\prime }\right)=\theta_{3}^{2}\exp\left(-\frac{{\left(x-x^{\prime }\right)}^{2}}{2\theta_{4}^{2}}-\frac{2\sin^{2}\left(\pi \left(x-x^{\prime }\right)\right)}{\theta_{5}^{2}}\right)$$ (6)

We considered an additional source of unstructured randomness through the term $\epsilon_{i}∼N\left(0,\sigma^{2}\right)$. We performed Bayesian inference (Markov chain Monte Carlo) over the joint distribution parameters $\left(\theta ,\sigma^{2}\right)$ and death counts for each time period of the 2020 year, based on 2000–2019 all-cause mortality data and suitable priors for the parameters. In the supplementary materials, we comment on more specific aspects and provide an extensive evaluation of our model.

#### IFRs

We deployed a hierarchical Bayesian joint model for reporting rates (and, hence, IFR) per age group (*a* taking values 0 to 40, 40 to 60, 60 to 80, and 80 +) and municipality *m*, collapsing over the temporal dimension. We infer the number of infected individuals (and, hence, IFR) based on reported cases *C*, positivity rates over time (*t*, month), municipality, and COVID-19–attributed deaths *D*. The main appeal of this framework is that although most of the components are not identifiable (e.g., if reporting rates and true cases are both unknown, the same observed case counts can be achieved by multiplying both by the same factor) (*37*), we can borrow from better-known quantities (e.g., rough estimates of prevalence, reporting, etc.) to enhance identification while propagating the appropriate levels of uncertainty over the parameters.

Specifically, the reporting rate $r_{m,t}$ links to the observed positivity rates $pos_{m,t}$ (in log-scale) through a logistic-linear relation (with parameters β), and we have included random effects $\epsilon_{m,t}$ to represent unobserved causes of reporting:

$$logit\left(r_{m,t}\right)=\beta_{0}+\beta_{1}\times pos_{m,t}+\epsilon_{m,t}$$ (7)

Total infections by municipality and age $I_{m,a}$ are a fraction $p_{m}$ of the total population $P_{m,a}$, that is

$$I_{m,a}∼Binomial\left(P_{m,a},p_{m}\right)$$ (8)

An implicit assumption in Eq. 8 is the existence of an underlying municipality-specific proportion infected $p_{m}$ so that on each age group, the number of infected people is (on average) $p_{m}\times P_{m,a}$. We also assumed the following relation for $p_{m}$:

$$logit\left(p_{m}\right)=p_{0}+\mu_{m}$$ (9)

where $p_{0}$ represents a baseline of the proportion infected and $\mu_{m}$ is a municipality-specific random effect.

We use parameters $\gamma_{m,t}\in \left[0,1\right]$ to represent the temporal spread of infections; $\sum_{t}\gamma_{m,t}=1$ so that $I_{m,a,t}=\gamma_{m,t}I_{m,a}$. Infections, cases, attributed deaths, and age-stratified population sizes are linked through a cascade of binomial models. We relate infections, cases, and reporting rates through

$$C_{m,t}∼Binomial\left(I_{m,t},r_{m,t}\right)$$ (10)

Infection fatality rates $IFR_{m,a}$ relate to infections and deaths through another binomial model

$$D_{m,a}∼Binomial\left(I_{m,a},IFR_{m,a}\right)$$ (11)

where the IFRs follow a stratified logistic-linear relation with SES and age mediated by parameters α, η, δ:

$$logit\left(IFR_{m,a}\right)=\alpha_{0}+\left(\alpha_{1}+\eta_{a}\right)\times SES_{m}+\delta_{a}$$ (12)

A comprehensive explanation of this hierarchical Bayesian methodology, including a discussion of its assumptions and several sensitivity analyses, appear in the supplementary materials.

## Supplementary Materials

science.sciencemag.org/content/372/6545/eabg5298/suppl/DC1 Materials and Methods Supplementary Text Figs. S1 to S59 Tables S1 to S4 References (*44*–*62*) MDAR Reproducibility Checklist
